# Exercise and Cancer Survivors: Lessons Learned from a Multi-Faceted Model for Exercise Prescription

**DOI:** 10.3390/jfmk3030038

**Published:** 2018-06-29

**Authors:** Laura Stefani, Francesco Sofi, Simone Magro, Gabriele Mascherini, Cristian Petri, Giorgio Galanti

**Affiliations:** 1 Unit of Sports Medicine, Department of Experimental and Clinical Medicine, University of Florence, 50141 Florence, Italy; 2 Unit of Clinical Nutrition, Department of Experimental and Clinical Medicine, University of Florence, 50134 Florence, Italy

**Keywords:** cancer, lifestyle, exercise prescription

## Abstract

Cancer is the second leading cause of death, and the most common diagnosis among the general population is breast and colon cancer. Recently, an increasing number of new cases of invasive breast and colon cancer have been estimated, and more people die from these diseases. In addition to the genetic pattern, diet and lifestyle including smoking, alcohol consumption, and sedentary behaviour have also been identified as potential risks factors. Recent studies of cancer survivors have shown the beneficial effects of regular physical activity to reduce the prevalence of comorbidity, muscle atrophy, weight changes, reduced aerobic capacity, fatigue, depression, and reduced quality of life. Dedicated and individual programs are crucial for achieving the goals of improving quality of life and reducing comorbidities. A multidisciplinary approach is fundamental: lifestyle assessment, including estimating the level of physical activity, as well as nutritional habits, may be the first step. A periodic cardiovascular examination is crucial for detecting asymptomatic early myocardial failure. According to current ACSM guidelines, different levels of exercise (low-moderate 40% and moderate up to 60% of the maximal HR) may be prescribed, and patients enrolled may follow the exercise program if in the absence of contraindications. The current paper reports observations from our clinical practice and provides practical strategies that bridge contemporary, published guidelines into practice within a multi-disciplinary team working with cancer survivors in Italy.

## 1. Introduction

Cancer is actually the second cause of death according to the WHO. An increasing number of subjects is expected to die as a result of this disease in several countries [1]. In Italy, epidemiological data for the period 1970–2010 show 250,000 new cancers diagnosed each year [2]. Among women, breast cancer is a quarter of all cases, while for men, the lung is now the most frequent cancer site. At the same time, the prevalence of colorectal cancer analysis in different areas of Italy shows that it is globally high for the centre and the northern part (200/100,000), while lower (75/100,000) in the southern areas. It is known that 70% of patients diagnosed with colon cancer within 5 years require treatment for cancer, while 13% require treatment for distant metastasis [3].

Numerous updated prevalence data are crucial for setting priorities in health care management and for developing a cancer control program. The onset of disease duration and age should be the basis for planning research and improving the quality of life of cancer survivors. The evidence also shows that this disease continues to become more chronic as a result of population aging and improvements in treatment, so an increasing number of cancer survivors in Italy should continue to increase. The recent literature highlights the role of physical activity for cancer prevention, particularly in cases of colorectal and breast cancers [4], while a probable and possible effect has been demonstrated in cases of prostate, lung, and endometrial cancers. It has been estimated that the risk reduction is approximately 25% up to three times, mainly if regular physical activity is practiced [5]. Several biological mechanisms induced by physical activity are identified as beneficial effects to prevent and modulate cancer progression. Recently, an association between genes polymorphism, stress oxidative system, and lifestyle factors has been demonstrated [6].

Oxidative stress is a complex equilibrium in which the improvement of the concentration in the oxidizing substance and the reduction of the defence elements of the human body represent the main mechanisms for losing the redox system [7]. The expressions of strenuous stressful cells are ROS (Reactive Oxygen Species) normally called “Free Radicals”. It has been shown that oxidative stress induces a “cellular redox imbalance” that has been found present in various cancerous cells [8]. In this context, the effects of ROS generate mechanisms such as DNA mutation and oncogenic stimulation. More recently, a dual role of ROS, either deleterious or beneficial, is reported. The “opposite” character of ROS consists in an action delegated to a secondary messenger in the intracellular signalling cascades, which induces and maintains the oncogenic phenotype of tumor cells. In parallel, a particular ability to provoke cellular senescence and apoptosis that plays an anti-tumorigenic species has also been demonstrated [9]. If deselected, the last plasma level of ROS leads to diseases like inflammation, neurodegeneration, respiratory diseases, and cancer [10]. Aging is associated with an increase in ROS production in skeletal muscle causing oxidative modification of proteins, lipids, and DNA. Studies highlight various data regarding the possible positive or negative impact of physical exercise on oxidative stress. Although they are apparently in contrast to each other, showing the presence of both of these effects when practicing physical exercise, it has otherwise been shown that they depend on the maximum on the intensity, type, and level of exercise [11], and on the age of the subjects considered [12]. Acute or chronic exercise can modulate in a different way the gene expression of metabolic or antioxidant enzymes that induce or reduce the intracellular mechanisms that generate ROS [13]. Some other mechanisms are activated by physical exercise and involved in cancer inhibition growth during exercise, including the AMPK (5′ AMP protein kinase), which is an enzyme present in the muscles, liver, and brain tissues. It can induce a reduction in fatty acids and promote the use of the fatty acids. It can modulate the plasma level of insulin. This level of protein subunit has been shown to be particularly active in response to acute and chronic exercise [14]. 

Other mechanisms are related to a specific activation of protein. AICAR (aminoimidazole-4-carboxamide-1-β-d-ribofuranoside) is a special peptide that stimulates the weight loss. This protein is particularly involved in many beneficial effects on glucose and lipid metabolism [15].

## 2. From Guidelines to Clinical Practice

In the case of cancer, progressive sedentary behaviour produces the inevitable increase in various cardiovascular risks factors in addition to well-known side effects, such as poor exercise tolerance and muscle fatigue, often due to chemotherapy or related to disease progression. The effectiveness of regular exercise on well-being has been demonstrated in different types of tumors of varying severity of disease. The new American Heart Association and American College of Sports Medicine recommendations [16] highlight the need to be active, as the ability and condition of patients can allow. In this report, we describe what we have learned from our clinical practice and provide practical observations that synthesize contemporary guidelines into practical use.

The literature supports the role of moderate-intensity regular physical activity to maintain a proper lifestyle to improve quality of life and reduce fat accumulation in cancer survivors. The fat mass is in fact the site in which the inflammatory substances are produced and maintained with effects of tumor genesis [17]. In this context and considering that quality of life is indeed one of the most important targets for cancer survivors, a basic lifestyle examination can be proposed as a first approach to establish “exercise on prescription program”. Except for very few conditions, such as extremely impaired blood cell counts and days when cardio- or nephrotoxic drugs are administered, physical activity should be recommended in cancer, especially for breast and colon cancer, in which the expectation of life is longer than normal compared to other types kind of cancers; a program for a lifestyle reconditioning is particularly important [18]. 

There is some evidence that physical activity performed before or after cancer diagnosis is related to reducing the risk of mortality among breast and colorectal cancer survivors. Other important evidence supports the role of physical activity, including active leisure physical time. There is evidence that being physically active before being diagnosed with cancer is predisposed to biologically less aggressive tumors [19]. Furthermore, pre-diagnosis physical activity can positively affect the treatment process, because it leads to a better functional ability to tolerate and complete the surgery and adjuvant treatment [20]. In summary, there is some evidence that physical activity before or after cancer diagnosis is associated with a statistically significant decrease in the risk of total mortality and cancer mortality among survivors of breast and colorectal cancer.

Starting from this background, this article proposes, on the basis of the data available from recent literature, the program of physical and practical exercises, aimed at analysing lifestyle and improving spontaneous and planned physical activity in a population of cancer survivors (breast and colon cancers) without any acute clinical contraindication to regularly exercise.

## 3. Exercise Prescription: Clinical Approach and Practical Protocol

According to recent literature, particularly the ACSM and AHA guidelines [17,18], some aspects of exercise as a prescription in cancer have been identified as potential basis for the present plan. The program is usually addressed to cancer patients in stable conditions and among these, as a consequence of the potential long-term life expectancy, subjects suffering from breast and colon cancer, without evidence of metastasis, are proposed as potential subjects to treat. In the first line, patients are initially enrolled after an initial check during the oncological clinical evaluation. Subsequently, the oral consent and the empowerment of the subject to the program are obtained.

The first step in our program is based on completing an assessment of the participants’ lifestyle, including the evaluation of nutritional aspects and the analysis of the amount of spontaneous and programmed physical activity level practiced. All data can be monitored by a validated questionnaire or, if available, by an accelerometer placed in the patient’s life for at least 7 days [21]. Notably, the questionnaire is often limited for self-reported physical activity, and the detection of the daily amount may be underestimated [22]. The authors report that despite the fact that the self-reported physical activities are evaluated by a specific interview that includes a series of question, the comparison between the two methods shows a very low correlation (*r* = 0.27). Some discrepancies in estimating actual physical activity may be due to a correct interpretation of the terms such as “physical activity”, “leisure-time”, and “moderate-intensity”. It is possible that some household activities may be interpreted as moderate intensity while they may be of low intensity [23].

To the contrary, we have observed that accelerometers do not accurately measure the true levels of physical activity as a consequence of the fact that monitors are generally worn on the hip and may lose the assessment of upper body activities. Furthermore, physical activities, such as cycling and many household activities, are probably underestimated by accelerometers.

In our experience, despite the acknowledged limitations of the questionnaires, the overall educational impact in the reconditioning of one’s lifestyle seems to be high and in the long run will produce a significant improvement in the daily habits that lead patients to pay attention to their amount of activity, playing an educational role. 

We have found that these procedures allow clinicians to review and verify daily levels of physical activity and then plan moderate intensity training programs. The prescribed exercise prescribed is a mixed exercise in which the aerobic component, such as fast walking and resistance, in which at least 8 muscle groups are involved, is represented.

### 3.1. Lifestyle Questionnaire

The first investigation for patients consists of a questionnaire composed by several questions focused on daily physical activity, including recreational activity and spontaneous physical activity represented by time spent walking for a daily activity or planned activity [24]. In particular, for cancer patients, the Karnofsky scale [23,25,26] can be used to better assess the stage of the disease and the daily habits of the patients. This information obtained can be combined with the general questionnaire by asking what kind of daily activity the patient conducts and how “free time” and “working hours” have been passed. Considering that physical activity represents a quantity of energy expenditure, this survey is very important for objectifying the level of physical activity level in order to graduate physical activity as “low, moderate, or intense” and classify the subjects as “active, mild, active, or sedentary”. Comparing this indirect calculation to the concept of Metabolic Equivalent of Task (MET), the three levels correspond to a different levels of intensity of physical activity: <2METS; >2 <3METS; >3METS, respectively. Dietary habits and overall nutritional status should also be explored during lifestyle assessment. The questionnaires are formulated as a daily diary in which the main nutritional habits have been reported for at least one week per month. The data are subsequently interpreted following the guidelines of INRAN (Istituto Nazionale di Ricerca per gli Alimenti e Nutrizione).

### 3.2. Nutritional Investigation

Correct nutrition is a protective tool in cancer to maintain weight balance [26]. Some special aspects concerning the quality of food can be evaluated and more precisely processed using dedicated software such as “WinFood” program (Medimatica-Italy). Parallel to a questionnaire, a food atlas is allowed, in which direct views of the size of the main course are possible for an easy estimate of the total daily meals as energy intake. The calculation of the Energy Balance, calculated as the net difference between the caloric intake and the energy lost, is therefore possible via the integration of the diet questionnaire data and the accelerometer report using a formula included in the dedicated software. In recent decades, with the aim of identifying a method to estimate the adherence to this beneficial dietary pattern, research has been focused on summarizing the diet through a single index or score resulting from the combination of included food components. Many indirect indexes have been proposed. Recently, we developed a novel adherence score targeted at giving practitioners a practical, easy, and evidence-based-instrument for assessing the adherence to Mediterranean diet at the subject level [27,28,29].

### 3.3. Accelerometer for the Physical Daily Habits

According to the literature, the pedometer or the accelerometer are included in this practical program as an easy system to investigate the free living physical activity [30,31]. Despite the differences between the diverse accelerometry, the various systems offer the possibility of an immediate visualization of the daily PA identified in spontaneous (SPA) and programmed (PPA) is possible. The accelerometer can be positioned on patient’s belt. It is normally worn for almost 7 days, excluding the night. After this period, the data obtained from the registration are downloaded to a laptop computer for the analysis of the SPA. More than 3 h a week is considered the point at which to distinguish sedentary from active subjects. Many other parameters are analyzed: the Physical Activity Level (PAL: definite as EE/resting Energy Expenditure), the average daily distance, the intensity of the PA expressed as slow (up to 3 km/h) or fast (up to 5 km/h) walking, and the number of total daily steps (Table 1). The daily PA can be further classified as “low walk” (LW), “fast walk” (FW), and jogging. As a consequence of the accelerometer report, these peculiar parameters will be collected and compared with data from the previous report for each patient [32].

### 3.4. Body Composition and Anthropometrics Parameters

As reported in the literature and supported by the ACSM guidelines and annual report committee, the body compositions parameters are normally measured at the beginning (T0) of the exercise program using a Bioelectrical Impedance Analysis (BIA-Akern/STA/BIA–Italy). The assessment is normally repeated every time the patients are investigated for a new updated program. The parameters generally evaluated include Fat Mass (FM), Fat-Free Mass (FFM) expressed in kgor as a percentage of total body weight, body water distribution including Total Body Water (TBW), Extra Cellular Water (ECW), Intra Cellular Water (ICW), and the Angle Phase (AP). The anthropometric parameters considered are the (BMI) Body Mass Index, the Waist Circumference (WC), the Hip Circumference (HC), and the ratio between them. The same parameters are also investigated after a period of at least 2–3 months of scheduled exercise.

### 3.5. Morphological and Functional Heart Evaluation

In order to verify the exercise tolerance of enrolled patients, myocardial function is normally evaluated by a standard 2D Echocardiographic examination performed according to the ASE guidelines [33]. The potential cardiotoxicity due to the previous chemiotherapic treatment represents the most important reason for paying attention to the myocardial performance, especially during exercise program.

This step is important, at least at the onset of the program, mainly in this particular type of subjects in which the heart performance can be influenced by the therapy and the severity of disease. It is also essential to exclude the presence of cardiovascular disease before starting regular physical activity. Moreover, effective daily physical exercise can be established by a Cardiopulmonary Test. The test calculates the maximal effort and therefore the corresponding Metabolic Equivalent of Task (MET) as an expression of the energy cost of physical activity. Alternatively, a cycloergometer test or a simple “6-min–walking-test” can be used for the estimation of the Energy Expenditure (EE) and Heart Rate (HR) to calculate the amount of the PA to prescribe. The option derives from the different levels of fatigue and exercise tolerance. The programmed physical exercise is normally established for up to the 60% of the VO_2_max at least 3 times a week [34]. The duration of the exercise is calculated by the Energy Expenditure parameter (Time = 150 kcal/EE).

### 3.6. Physical Exercise Plan

At the beginning of the program, the type of initial exercise that is proposed to improve the quality of the life style normally includes an aerobic component such as fast walking, moderate walking, jogging, or running. Resistance exercises consisted of a program with no additional weight to body weight. According to the guidelines of the American College of Sports Medicine, it includes 3 sets of 10 repetitions for each of the 8 major muscle groups (legs, hips, back, chest, abdomen, shoulders, and arms) [16]. Initially, subjects were instructed to aim for 2 sets of 10 repetitions for each muscle group and asked to increase the series and repetitions to 3 × 10 repetitions as the strength and resistance increased. Subjects were told not to exceed 3 × 20 repetitions. The flexibility exercises involving the calf muscles, quadriceps, and hamstrings consisted of a static stretching set in which the subject maintained elongation for at least one minute.

Aerobic training included 30 min of fast walking at 70% of their individual maximum level. The individual level of aerobic exercise was determined by the corresponding level of the perception effort estimated at 6′MWT and equal to the sixth point if compared to the CR10 reference efforts scale CR10 [35]. 

The intensity of these exercises is otherwise modulated as a consequence of the severity of the illness and of the effort tolerance. In relation to this aspect, it is possible to plan a more or less vigorous physical activity [36,37], identifying two other subgroups at different levels of exercise (Low-Moderate or Moderate). For the former, the intensity will be at least 40% of the maximal HR, and for the second, up to 60% [27]. This cannot be considered an effective progression of the physical charge, but mainly a range of activities to modulate the intensity of the exercise. The aerobic exercise training can be allowed inside, on treadmills or exercise bicycles and climbing machines under the supervision of expert training subjects, or outdoors with the same intensity. Patients are normally subjected to the exercise program for at least 10 weeks and periodically re-evaluated by a follow-up.

At least 150 min of physical activity per week is recommended. Adherence to the program is controlled by a periodic questionnaire that is submitted every month or at least every 6 months. Daily physical activity is declared inside.

### 3.7. Biomechanical Evaluation

Some biomechanical aspects are also investigated. The assessment consists of the strength and flexibility of the upper and lower limbs: the strength performance of the upper body is evaluated by the hand-grip test, while Chair Stand test is used for the lower part; the flexibility of the back and the ischiocrural muscles are evaluated by the Sit & Reach test.

The data were found in order to verify the progressive improvement of the function of the muscles and skeleton apparatus.

### 3.8. Psychological Analysis

The psychological aspect can be otherwise estimated by a dedicated questionnaire focused on symptoms or perception of life expectance [38].

The index of the psychological quality of life for cancer patients is normally studied by LASA (Linear Analog Self-Assessment). This score includes some variables (anxiety, depression, confusion, energy, and anger). All these aspects are considered at the beginning of the exercise program and during the follow-up. In any case, the possible application of SF36 model, to estimate the quality of life, can be useful.

### 3.9. Follow-Up

**Figure 1:**
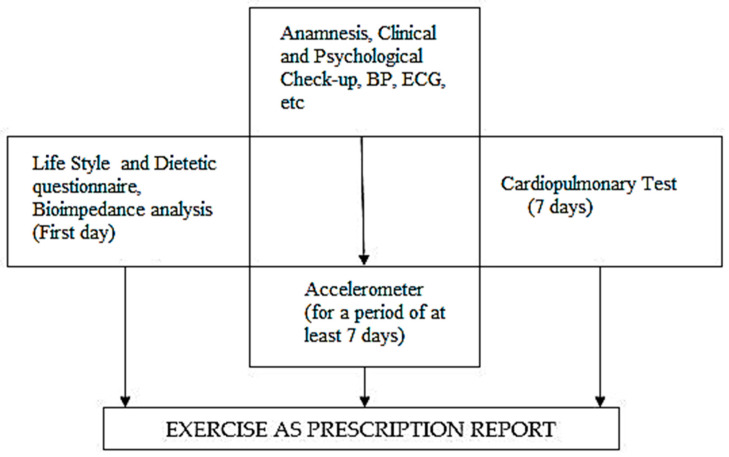
The figure shows the chronological sequence of the “exercise on prescription program”. The flow-chart shows the chronological sequence of the “exercise on prescription program”.

Follow-up is focused on the cardiovascular, psychological, and biological parameters. For the second specific aspect, cancer plasmatic makers are normally used to achieve better monitor disease progression. The regular follow-up, approximately every 2 months, including all the exams with the exclusion of the Cardiopulmonary Test is scheduled. (Figure 1) The results of the cardiovascular parameters obtained allow one to verify the adhesion to the program; the progressive improvement of spontaneous physical activity, the maintenance of weight within the normal range; and the absence of symptoms related to a possible reduction of exercise tolerance, recovery of tone, and strength of muscles.

## 4. Clinical Expectations

The expected results are linked to a global increase of the quality of life. They can be summarized in the following list:

(1) Decreased cancer progression, as a consequence of the expected positive effect of the exercise indirectly estimated from the specific plasma level of neoplastic markers and from the echocardiographic or tomography report.
(2) Progressive increase of VO_2_max and exercise tolerance.
(3) Reduction of the sense of fatigue.
(4) Improvement of body composition parameters, with an improvement in FFM.
(5) Reduction of the prevalence of edema with normal hydration of the body.
(6) Increase in muscle hypertrophy with consequent reduction of possible bone damage.
(7) Reduction of pain.
(8) Reduction of anxiety and depression.
(9) Improvement of quality of life, mainly for psychological aspects.
(10) Recovery of time dedicated to occupational and recreational activities, with progressive recovery of social habits.
(11) Reduction of therapeutic costs as a result of the overall improvement of quality lifestyle and reduction of bone or muscle injuries.

Considering the multiple parametric data, the follow-up can be correctly obtained by using quality of lifeanalysis. The SF36 questionnaire includes different sessions to detect general health, physical pain, vitality, social activity, the role of emotional behavior, fatigue, daily physical activity, etc. The other anthropometric parameters can be investigated by Bioimpendance analysis, and specific tests such as hand-grip and chair test. For the potential reduction of the social cost due to the improvement of the QoL in cancer, feedback regarding the reduction of the days of the disease could be important. 

## 5. Conclusions

As a result of better diagnosis and treatment, more people survive cancer. Cancer survivors are, however, affected by several physiological and psychological side effects including muscle atrophy, weight changes, reduced aerobic capacity, decreased strength, reduced flexibility, anxiety, and depression; therefore, a global reduction in quality of life is evident. Most of these side effects are controlled by regular physical activity [39]. Exercise is now considered an important tool for rehabilitation of cancer patients, as well as the ability of this to reduce several potential risks factors related to the incorrect lifestyle. More recently, some studies have demonstrated the positive effects of moderate exercise intensity for cancer survivors [39]. It has been established that an amount of “moderate daily physical activity”, between 65% and 80% of the maximal heart rate, can be useful for improving public health. According to international literature, a prescription program for the general population has also been proposed in Italy [40]. In particular, for cancer survivors, the ACSM guidelines have identified two different levels of exercise intensity that demonstrate low-intensity exercise health benefits such as those from moderate–intensity [41]. Some further studies have confirmed that this aspect shows that regular physical exercise it is not to be strenuous to obtain health benefits, and therefore in this context the improvement of lifestyle represents the first step towards starting adequately. However, the results of the exercise in long-term treatment beyond the 10-week duration of the exercise program are not yet fully clarified. This is one of the rationales for this proposal program, which includes cancer survivors without metastatic involvement and those suffering from cancers (breast and colon) most frequently associated with a long period of surveillance. To highlight the effectiveness of the exercise program, it is reasonable to consider it important to select two other subgroups, which are aimed at different intensities of exercise (low-moderate and moderate). This is a relevant to better appreciating the possible therapeutic impact of long-term exercise between different levels of disease severity. It is reasonable to think that if low-moderate exercise intensity is better tolerated, different effects could be achieved with different levels of physical therapy. The aim of this protocol is therefore not exclusively oriented to the practical therapeutic effects on quality of life, but also towards ascertaining which level has produced the greatest benefits. As consequence of this feature, the attendance rate for participants could be higher in the first than in the second program. To provide an optimal guide for the survivors, new professional figures will be employed in the program that will be prepared to understand the common physical therapeutic approaches in cancer. These professional figures will play an important role, mainly in the follow-up to the program for the periodical evaluation of the biomechanical and nutritional habits of the patients.

Most of the expected results will be correlated with a clear improvement of the quality of life demonstrated by the accelerometry report in which it is possible to objectively evaluate a progressive improvement of spontaneous physical activity and progressive complete adhesion to the exercise program.

At the end of this exercise, as a prescription program, a report with various information related to the exercise program will be made available to patients. A final written report constitutes an important summary of the patient’s exercise program and is an important link with their personal physician. 

## 6. Practical Implications

The recommendation to improve lifestyle in cancer survivors is the first step before starting an appropriate exercise as a prescription program.

Several clinical implications and benefits derive from this approach. Among these, one can expect a reduction in body weight, an improvement in psychological status, and, in addition, positive economic results due to a reduction in the side-effects of chemotherapy, especially if in presence of a great adhesion [42]. 

Although long-term follow-up of a physical activity program in cancer is not yet available, it is reasonable to think that a program to reduce sedentary lifestyle is less effective than a normal hospital service [43].

Patient empowerment and motivation are two crucial points for a successful lifestyle. For this reason, patient referral schemes have been widely promoted in addition to the accelerometer report, to supervise training sessions taking place indoors or in public leisure facilities [44,45].

When regular physical activity is allowed, it is possible to plan a full exercise on the prescription program. The economic evaluation of health through randomized controlled trials can be proposed alternatively to verify the cost-effectiveness and cost-utility ratios. In summary, regarding the new concept of exercise prescription, the correct prescription must be multiparametric. The effects of exercise may vary with the specifics of exercise intervention [46,47]. Detailed information is needed on the effects of the intervention (i.e., timing, duration, and delivery modality) and exercise-related characteristics (i.e., FITT factors) for patients with cancer in order to identify which prescriptions are effective to improve QoL and physical function.

## Figures and Tables

**Table 1:** Description of physical activity (hours, steps, METS, distance). The figure includes the parameters as distance, time, intensity, and Physical Activity Level (PAL) as expression of mean amount degree of daily physical activity. All these parameters are used to classify the lifestyle of the patients, just in case accelerometry is used.

**Physical Activity Level Expressed as Daily and Weekly Duration Time**					
**Physical Activity (in duration of time during 6 days)**					
Sedentary (hours)	3 METs (hours)	3–4.5 METs (hours)	Jogging (minutes)		PAL
**Physical Activity (in duration of time per day)**					
Sedentary (min)	3 METs (min)	3–4.5 METs (min)	Distance (m)	Steps	Calories Spent (kcal)
